# “Don't Gamble With Children's Rights”—How Behavioral Design Impacts the Right of Children to a Playful and Healthy Game Environment

**DOI:** 10.3389/fdgth.2022.822933

**Published:** 2022-05-02

**Authors:** Simone van der Hof, Stijn van Hilten, Sanne Ouburg, Max V. Birk, Antonius J. van Rooij

**Affiliations:** ^1^Professor of Law and Digital Technologies, Center for Law and Digital Technologies (Elaw), Leiden University, Leiden, Netherlands; ^2^Project Assistent Privacy and Ethics, Amsterdam University of Applied Science, Amsterdam, Netherlands; ^3^Privacy Consultant, Privacy Company, The Hague, Netherlands; ^4^Industrial Design, Eindhoven University of Technology, Eindhoven, Netherlands; ^5^Senior Researcher, Trimbos Institute, Utrecht, Netherlands

**Keywords:** gaming, behavioral design, children, children's rights, economic exploitation, play, health, data protection

## Abstract

Gaming is an important pastime for young people to relax, socialize and have fun, but also to be challenged, show creativity and work together to achieve goals. The design of games can have an impact on their behavior. With the changing revenue models of games, we see that game design is increasingly taking forms that do not always have a positive impact on children and may interfere with, or even violate, children's rights. This article examines how evolving revenue models of games impact user's behavior *via* game design. Behavioral design in games thus raises questions about children's rights to play and recreation, to health, to protection from economic exploitation and to data protection.

## Introduction

Gaming^1^ is a hugely popular online activity among teenagers and young children: a large percentage play games and the time they spend playing games has greatly increased during COVID (1). Games can be played *via* various devices, such as consoles, computers and mobile phones. Gaming can be a favorite pastime for many reasons: it can be challenging to conquer certain quests, a way to find distraction from the routine of everyday life or to meet friends. Gaming is, according to the UN Children's Rights Committee, a form of play that can be enormously important for children in their development. It is not only fun, but also very educational (2). The positive impact of gaming should therefore not be underestimated.

At the same time, we see that gaming can also have negative effects on gamers. In part, this negative impact is caused by choices made in the design of games, which direct or influence the behavior of gamers. This is also called “behavioral design” (3), a concept related to persuasive design (4), nudging and choice architecture (5). Behavioral design, however, is not necessarily negative. Examples of behavioral design having a positive impact are those that increase the joy of gaming through challenging, but doable, achievements without negatively affecting the well-being of the gamer. But there are also numerous examples of behavioral design not necessarily mediating a positive gaming experience. These types of behavioral design are called “dark patterns” (6), i.e., design choices that make the gamer do something that is in the game company's interest, usually because it allows them to make money, but not necessarily something the gamer wants to do themself or something that is in their best interests.

The emergence of dark patterns seems to be directly related to the emergence of innovative revenue models for games in recent years. Whereas, initially a game was bought in a box for a fixed amount in the shop, nowadays companies earn from games on the basis of a subscription, advertisements and micro transactions. These revenue models bring with them the need to steer behavior toward profitable actions to generate, and overall, maximize profit.

Gaming, as a form of play, can make an important contribution to the well-being and development of children. This is not necessarily the case with games that are driven by behavioral design with a view to profit maximization. In fact, such commercial practices are usually not in the best interests of the child and, moreover, may interfere with, or even violate, other children's rights, such as their rights to health and protection against economic exploitation. In addition, these forms of behavioral design can lead to unfair commercial practices which, in the case of children, should be given particular attention.

The purpose of this contribution is to explore how behavioral design in gaming impacts children's rights. In doing so, it is important that games, in the case of children, should be explicitly designed in a way in which their interests are a primary consideration. This means that games should not only not be harmful to children, but should also contribute to their well-being and development. The welfare and development of children is protected by more specific children's rights as laid down in the UN Convention on the Rights of the Child 1989 (further: UNCRC). In relation to behavioral design and children's welfare, the rights of children to health, play, data protection, and protection against economic exploitation are particularly relevant. The relevance of these rights will each be explained from the perspective of behavioral design. Before doing so, we will first give a brief explanation of the concept of behavioral design and the related dark patterns.

## Behavioral Design in Games and Revenue Models

With the term behavioral design we mean the design of digital (video) games in its various manifestations (console games, PC games, mobile games etc.) that influences the behavior of the gamer. Behavioral design can have both a positive and a negative impact on gamers. In this respect, behavioral design is meant as a neutral term that in itself says nothing about the effect of design on gamers, only that there is an intent to influence behavior through design. Examples of positive behavioral design are, for instance, encouraging gamers to exercise and go outside [e.g., with Pokemon Go (7)] or by encouraging teamwork (8) or societal engagement (9) through gameplay.

However, there are also numerous examples of behavioral design having a negative impact on the gamer. Some examples are: continuous notifications to make a person return to a game even when they don't want to, or making it difficult to stop playing, which can interfere with other activities such as school, sports and meeting up with friends. These effects may be stronger if the gamer is particularly vulnerable in some way, for example due to a problematic personal situation, psychological vulnerabilities, or because the gamer is vulnerable in terms of development. The latter may be the case with children, for example, although here too there may be a combination of factors (e.g., also problems at home, social problems or mental health problems) that can occur with gamers more generally.

Although we use the neutral term behavioral design in this contribution, the focus will mainly be on the negative effects of behavioral design. The reason for this is that with the evolving revenue models underlying games, we are seeing changes in game design that are not necessarily in the interests of the gamer or even harmful to them. In this context, we are referring to so-called dark patterns as specific forms of behavioral design that can manipulate and mislead the gamer. Children can be even more susceptible to these forms of manipulation, although the fact that they often work with recognizable behavioral change principles and methods means that, in principle, any person can be influenced by them.

Zagal et al. define dark patterns as “intentionally used by a game creator to cause negative experiences for players and against their best interest” (10). Dark patterns thus consist of design choices that make the player do what the company wants to e.g., stimulate profit making instead of letting the gamer follow their own preferences. In other words, the gamer is manipulated and cannot make informed decisions. Based on Zagal et al.'s (6) work, we distinguish the following categories of dark pattern:

- **Temporal dark patterns**: let gamers play longer than they want to, e.g., by letting them perform repetitive or tedious tasks (grinding) or imposing timed events on the gamer in order to be successful. Furthermore, the expectations of games regarding the time investment can be unclear to gamers, as well as what it takes in terms of time investment to become an experienced gamer in the game.
- **Monetary dark patterns**: trick the gamer into spending more money than they want to by using deception or covert strategies, for instance by pushing to pay for game progress by reducing gamer's abilities or making progress otherwise difficult (friction) (e.g., pay to skip), making successful game play dependent on extra content or increasing the chance of winning by investing extra money in the game (e.g., pay to win).
- **Social capital-based dark patterns**: use the gamer's social relationships to benefit the company's interest (e.g., impersonating friends);
- **Psychological dark patterns**: use (other) psychological tricks to let the gamer make decisions they don't want to or are not in their interest (11).

These forms of dark patterns are not mutually exclusive and can reinforce each other. Some are blatantly and overtly manipulative, others move in the direction of more established marketing techniques. Moreover, behavioral design can also be used for good, by e.g., setting limits on the (excessive) investment of time in games and encouraging positive social and financial behavior.

Given that dark patterns are aimed at getting as much time and attention from the gamer as possible, and inducing them to make substantial financial investments, there seems to be a direct relationship between the use of dark patterns and the revenue models of games. In the box-sale model of gaming, i.e., offering games-as-a-product, the focus can be on the enjoyment of the game and strategies to entice the gamer to buy or engage in marketing are not necessary. The gamer pays for the game up front and is not confronted with, for instance, in-app purchases required to continue playing (quickly) or with advertisements that must be viewed every so often before he or she can continue playing. This has been changed by new revenue models that rely on new strategies to make money from games, which include game monetisation models such as subscription models but also pay to play, free-to-play, and play to win, many of which use, among others, microtransactions (in-app purchases, loot boxes), season passes, in-game (video) advertising, product placement. Incidentally, even if you pay a substantial amount for the game up front, microtransactions may still be needed to enjoy the game fully. An example is the Battlefront 2 controversy, where gamers had to pay extra for certain popular characters, something that has been reversed after massive criticism (12).

Recently, data-driven models have also come into prominence, in which the vulnerabilities of gamers can be exploited in a more targeted way, for example through personalized marketing (13) and linking players in such a way as to achieve the most profitable match (so called monetised matchmaking) (14). Data-driven models are driven by gamers' behavioral data, so-called data given off, from which new knowledge (inferred data), including gamers' characteristics and interests, can be generated. Inferred data can include particularly sensitive information about individuals, information they may not wish to share, such as gender, sexual orientation or medical condition (15). Apparently, the technology can now be refined in such a way that it is possible to target a single person on the basis of characteristics (so-called nanotargeting) (16).

Both the emergence of new monetisation strategies and the consequent development of dark patterns to influence gamers' time, attention and spending patterns in relation to gameplay result in an impact on children and their rights as enshrined in the UN Convention on the Rights of the Child 1989.

## Behavioral Design From a Children's Rights Perspective

In 1989, the UN Convention on the Rights of the Child (hereafter: UNCRC) was adopted and in 1990 it entered into force (17). The UNCRC was ratified by every country in the world, except for the US, making it the most successful international treaty in that respect. The Convention recognizes that children, rather than objects of protection, are subjects with rights, but in view of their development they deserve special attention, including special (implementation of) fundamental rights and specific protection in line with their evolving capacities.

In the following sections, relevant children's rights from the UN Convention will be analyzed in the light of behavioral design in games. Design can be understood broadly and also includes terms of use, community guidelines etc, however here we will focus on behavioral game design specifically. We start with a right that is also one of four fundamental principles of the Convention: the right of children to have their best interests taken as a primary consideration with respect to activities that affect them (Article 3 UNCRC). Then we will look at more specific rights, namely the right of children to play (Article 31 UNCRC), the right to health (Article 28 UNCRC), the right to protection against economic exploitation (Article 32 UNCRC), and the right to data protection (Article 16 UNCRC).

### Designing Games in the Child's Best Interests

In all activities with an impact on children, the best interests of the child must be a primary consideration (Article 3 CRC). When gaming companies create a game that is also played by children, the best interest of the child principle should be considered. For this, it is necessary to first determine what impact the game has on children. This concerns both the positive and negative impact on the development and welfare of children. This exercise is also called a child impact assessment and it is not a one-off activity (18): games are usually developed further after release, for example by incorporating new or different monetisation strategies, which can change the impact. Moreover, the impact of games may only become clear when they are played. The best interest of the child must therefore be a constant consideration during the design of games, from the moment the idea is first conceived and throughout the life cycle of a game when it is further developed by engineers and used by the players. To make this practically possible, we suggest that the best interest is part of evaluation of the game at every major milestone, e.g., when the core loop of the game is defined, during the choice of the theme of the game, when the monetizing model is introduced, and during all release versions of the game. By frequently evaluating the best interest of the child, unintended or even undesirable uses or consequences with respect to children can in theory be dealt with when games are further developed. In practice, however, it can be difficult to make changes to a game's design at a later date; while appearances (e.g., the color of a particular in-game item) may be easy to change, many elements in a game are often systematically related in the sense that they are part of the game play (e.g., rewards systems, of which loot boxes^2^ can be a part) and cannot simply be removed without having a wider effect on the game. This means that game developers should preferably try to estimate the impact and age appropriateness of a game at a very early stage.

In this context, it is also relevant that research into the impact of gaming and game design is in development and may provide new insights that can lead to the adaptation of game design if it is not in the best interest of children or even harmful to them. In addition, with respect to children it is advised to apply the better safe than sorry approach (precautionary principle), which means that even if there is insufficient hard evidence, it is better not to choose a particular design if it may have a negative impact.

In the broadest sense, the best interests of the child means that activities that have an impact on children must ensure the child's well-being and development (18). It is therefore not enough merely to prevent harm or negative consequences to children, although in gaming, the focus is often on the potentially harmful effects. The positive side of gaming is just as important to include in an impact assessment. The best interests of the child also include providing children with a meaningful and fun online experience that can make an important contribution to their development. Children should also not be excluded or deprived of a gaming experience just because their interests and rights require special attention in the design and development of games.

Moreover, a balance must be struck between children's protection rights and their participation rights, such as their rights to development, freedom of expression and freedom of information. Ideally, games contribute to the child's well-being, participation and development, while preventing harm. An example where that does not go entirely well is Pokemon Go which encourages players to exercise and socialize more (positive impact) but also brings players to physical locations at, for example, late hours where they are a target for theft and assault (negative impact).

Additionally, what is good for the well-being of some age groups is not necessarily good for other age groups. This is related to the evolving capacities of children (Article 5 CRC). “Evolving capacities” is a concept put forward by the CRC that acknowledges that age matters for the interpretation and implementation of children's rights, for example, in the sense that as their competencies grow there is less need for protection and more reliance can be placed on their ability to make their own decisions (19). Hence, the impact that games can have on children depends, among other things, on the age and development of children. For this reason, tools such as content classification (e.g., PEGI), privacy-friendly age verification and, in the case of younger children, safety tools to be used by parents are also worth considering. In any case, it is important to keep in mind that, whenever a game is played by children, game design is inextricably linked to the best interests of the child through the content and contacts they may encounter, the functionalities of the game that direct their behavior, and the terms and conditions that impact their rights. Moreover, these factors can be interlinked by the underlying and evolving business models of games as addressed.

The best interests principle is also inextricably linked to the right of children to be heard (Article 12 CRC) (18) because in order to find out what their interests, expectations, wishes and concerns are one has to find out their views on gaming and game design. As a recent study by Livingstone and Pothong shows, children have particular wishes and concerns in relation to gaming, some of which also pertain to game design (20). It is essential that game designers involve children in the design of games and learn from both their positive and negative gaming experiences. As far as we know, game studios engage in experience testing after development but with a few exceptions, but there is no direct participation of children in the design of games, and obviously it also requires special expertise to co-design with children making it a challenge that would perhaps rather be avoided or simply forgotten.

In connection with the best interests of the child, it is relevant that children are considered vulnerable consumers because of their age, evolving capacities (Article 5 CRC) or credulity can make them particularly susceptible to particular commercial practices. Their capacities in, e.g., recognizing and understanding online advertising and commercial content, will vary greatly from one child to the next depending on age and maturity (21). Due to emerging business models and especially the enormous economic importance of in-game monetisation, the significance of protecting gaming children as (vulnerable) consumers has become much more relevant. When assessing the (un)fairness of commercial practices the impact on children will be assessed from the perspective of the average member of the group of children in question (Article 5 Unfair Commercial Practices Directive). In the case of a game developed for children or teenagers, the average child of the relevant age group will be the benchmark (22). Extra protection for children is needed when games are specifically aimed at children. This is certainly the case when it is reasonably foreseeable that a game is likely to appeal to children, e.g., through its content, style and/or presentation (22). A significant determinative factor is whether children are known to play the game, or if the game is marketed to children. The use of cartoon-like graphics, bright colors, simplistic gameplay and/or language could be an indication that a game is likely to appeal to children (23). Similarly, children are seen as a vulnerable group when it comes to the processing of their personal data. It is generally accepted that children are less able to assess the risks and consequences of data processing and less aware of safeguards and rights that can help protect their personal data (24), where children deserve more protection. The fairness of commercial practices and data protection will be addressed in Section Avoid Exploitative Game Design and Limits to Data-Driven Game Design, respectively.

### Playful Game Design

Gaming is an important pastime for young people to relax, socialize and have fun, but also to be challenged, show creativity and work together to achieve a goal. Gaming immerses a person in another world, similar to reading books or watching films, but in an active way. Gaming as a form of play and leisure is thus linked to one of the perhaps least known-but no less important-rights of children: to be able to play and relax in a way that is age appropriate (Article 31 CRC). A right that intends to contribute to the optimal mental, social, cognitive and physical development of children which expresses their best interest in a way that is inseparable from what it means to be a child and to grow up by being given time for playful activities free of obligations.

The importance of play cannot be underestimated as the following quote from the Children's Rights Committee illustrates:

“*Play and recreation are essential to the health and well-being of children and promote the development of creativity, imagination, self-confidence, self-efficacy, as well as physical, social, cognitive and emotional strength and skills. They contribute to all aspects of learning; they are a form of participation in everyday life and are of intrinsic value to the child, purely in terms of the enjoyment and pleasure they afford. Research evidence highlights that playing is also central to children's spontaneous drive for development, and that it performs a significant role in the development of the brain, particularly in the early years. Play and recreation facilitate children's capacities to negotiate, regain emotional balance, resolve conflicts and make decisions. Through their involvement in play and recreation, children learn by doing; they explore and experience the world around them; experiment with new ideas, roles and experiences and in so doing, learn to understand and construct their social position within the world.” (at p. 4)* (18).

From the perspective of gaming experiences that contribute to children's development, play is an activity that makes a hugely important contribution to children's development (18) and we see this potential for development reflected in gaming. Depending on the game, children can learn to cooperate, meet new people, socialize with friends, develop their identities, develop or improve certain skills or practice a foreign language (usually English) (25). Developing skills include for example improving one's reflexes in fast paced shooters such as Counter Strike Global Offensive. One study found, e.g., that “Playing action video games—contemporary examples include God of War, Halo, Unreal Tournament, Grand Theft Auto, and Call of Duty—requires rapid processing of sensory information and prompt action, forcing players to make decisions and execute responses at a far greater pace than is typical in everyday life” (26). Games can also be instructive because they are developed from a cultural, artistic or historical perspective. Games such as Oregon Trail^3^, We. The Revolution^4^, and Europa Universalis^5^ are historically accurate, to a certain degree, and can contribute to a deeper understanding of history. There are also political simulation games such as Democracy 4^6^, which can contribute to a better understanding of real-world political struggles and perspectives. Other games such as Shenzhen I/O^7^ and Screeps^8^ contribute to learning programming by effectively teaching and requiring knowledge in the programming languages assembly and javascript, respectively. A plethora of games also increase a child's artistic capabilities by enabling them to build certain structures. Gaming may therefore support a child's right to education (article 28/29 CRC), although education should not be an end in itself in play.

Play, also in the form of gaming, cannot therefore be optional but must be recognized as an essential part of childhood. And play must meet a number of criteria in order to be distinguished as such from other activities. According to the Children's Rights Committee, play is free, self-determined time in which the child has control over the course of the activities and is driven by intrinsic motivation (18). Livingstone and Pothong have extended the list of essential characteristics to 12 qualities of free play, based in part on the insights of children themselves: free play is (1) Intrinsically motivated (2), Voluntary (3), Open-ended (4), Imaginative (5), Stimulating (6), Emotionally resonant (7), Social (8), Diverse (9), Risk-taking (10), Safety (11), Sense of achievement (12), Immersive. These qualities of play form a benchmark for forms of gaming that take into account the right to play in an age-appropriate manner and should therefore also be a guiding principle for age-appropriate game design (20). Age appropriate game design that can be considered free play then means that features that contribute to a positive gaming experience of children should be encouraged and features that lead to a negative impact should be avoided. A recent model of playfulness that can also contribute to playful game design distinguishes four basic components in which playfulness can be expressed: Other-directed, Lighthearted, Intellectual, and Whimsical (27). Other-directed again looks at the social aspect of games while playing where the focus is on enjoyment in playing with others, including e.g., role-playing. A game is light-hearted if you do not have to worry too much about the consequences of your behavior, even if risky, and playfulness is a natural part of daily life. Intellectual playfulness can be stimulated by having to solve specific tasks and puzzles and thus a sense of achievement. Whimsical is about being able to enjoy strange situations and be weird.

Positive game experiences can be evoked through the design of what is generally understood to be non-scripted play (4) which includes open ended forms of gaming that provide children with agency and allows creativity to be given free rein while playing. In the case of gaming, one can think of Minecraft^9^ or Terraria^10^, where (unless, for example, private servers with timed events are involved) children have a great deal of freedom to shape their gaming experience themselves. Non-scripted play resonates with many of the qualities of free play put forward by Livingstone and Pothong. Interestingly, risk-taking is put forward as a characteristic of free play by children themselves (20). This may seem to be at odds with safety, but that is not the case. Risk-taking-if it takes place in a supportive and reasonably safe environment-can actually contribute to making children more resilient by teaching them how to deal with risk and teens may even like seeking risks which is giving them a sense of freedom and autonomy (28). Such risk-taking in gaming can involve engaging with particularly challenging experiences following from game design, such as being outside at night in Minecraft or surviving in battle royale games. Challenges can also be economic in nature, such as in the form of gambling elements. We will return to this later in this article but as a rule these are seen as problematic for children and young people more generally.

There are negative gaming experiences due to game design that should be avoided as much as possible in view of play that contributes to children's well-being. It is recognized that getting enough rest from activities and adequate sleep are an important part of the right to play and leisure. This is in contrast to design features that constantly disturb players with notifications or push them to keep playing in order to be competitive. Research among UK 6 to 17 year olds shows that 69% find it difficult to stop playing even if they would like to (20). Moreover, although gaming can also be seen as an adult-organized form of recreation that can contribute to the development of children, in order for it to be free play participation should be voluntary (18). Games don't qualify as such if there is little or no freedom to shape the gaming experience (so-called scripted play). The question is to what extent there can be voluntary play if the design of games contributes to (or at least does not protect against) obsessive gaming or has sticky features that make it difficult to get away from the game. Here, in addition to the design, the personality and vulnerability of children or young people play a role. In any case, game design that causes negative experiences against the gamer's best interest, e.g., through manipulation, defies the autonomy of the gamer to play (and stop playing) freely. To change this, gamers can be given more control over the gaming experience with settings that let them decide how they want to interact with the game. For children, these settings should be pre-set to the least intrusive standard (e.g., notifications are off by default). Many of these design features that lead to negative experiences are often inextricably linked to the revenue models of the gaming industry discussed earlier. Such design therefore interferes not only with children's right to free play but also with their right to protection against economic exploitation, which we address in Section Avoid Exploitative Game Design when discussing exploitative game design.

Furthermore, the right to play also presupposes inclusivity, which means, among other things, that gaming should also be accessible to children with disabilities and that games should steer clear of stereotyping, prejudice and discrimination, and be respectful of gender, ethnicity and, in general, the vulnerability of children (29). The right to play here must be considered in connection with the right to non-discrimination (Article 2 CRC). However, the gaming environment is not always inclusive and this is therefore a point of attention and can even be seen as an opportunity when designing games (responsible and inclusive design) (25). In the same vein, the gaming environment is not always perceived as safe by children. Game design can, for example, aim to encourage gamers to make as many contacts as possible to contribute to game engagement, but in the case of (younger) children it is preferable to close that kind of setting by default (e.g., a profile set to private by default).

Given that play, including gaming, can and should make an important contribution to the development of children, there is an important relationship with setting conditions for healthy game design.

### Healthy Game Design

Designing games in a way that is in the best interest of children and thus contributes to certain characteristics of child friendly playful experiences, as discussed in the previous sections, requires adding another important factor: gaming should also contribute to the healthy development of children-or at least not harm their health. Children's right to health is recognized in Article 24 CRC and includes many aspects such as the importance for the development of children to engage in healthy behaviors (30). Gaming can contribute to children's development as we have seen previously by e.g., allowing them to develop their personalities, social networks and skills. For instance, gaming can train their reflexes and coordination, improve their learning skills, increase their socialization and teamwork skills.

However, game design can also have a detrimental effect on the health and well-being of children. Children and young people may even be more susceptible to the negative effects of some forms of game design because they are still developing. Therefore, from the perspective of the child's best interests, it is essential that these effects are properly assessed and that their specific vulnerabilities are taken into account by avoiding negative effects of game design. More specifically, the right to health includes the prevention of health-related harm. Tools such as the digital balance model from Netwerk Mediawijsheid and Trimbos Institute can, for example, help gamers reflect on their game use in relation to their mental, social and physical health (31, 32).

Negative impact on gamers' health can occur in several ways or be caused by specific game experiences, such as through stress, demands, social exclusion, social harm (e.g., invasion of privacy, hate speech or cyberbullying, all of which may also result in mental harm), mental harm (e.g., sexual abuse or aggression from playing violent games), physical harm (lack of exercise, obesity, poor sleep) (18). Again, the game providers will therefore have to take into account the avoidance of harm in any way through design, terms of use and community policing instruments. However, it also means more specifically that behavioral game design that leads to unhealthy behavior or health harm should be avoided. At least that the gamer should have the ability to control settings in ways that make the game environment more pleasant for them, settings that for children are by default tuned to the most healthy features.

In this respect, it is again important that game design takes into account the evolving capacities of children in order to ensure a healthy development of children of all ages (19). For adolescents, this may have a different outcome than for younger children. What is harmful for younger children may not be so for adolescents, which is e.g., reflected in the age classification of games by PEGI. The evolving capacities are also important in determining the extent to which behavioral design choices have a detrimental impact on children at different age and developmental stages.

Specific health concerns include game design that leads to excessive gaming or even game addiction. Although a direct link cannot be proven, it is noteworthy that subscription-based gaming was associated with a growth in problematic gaming behavior and gaming addiction reports (33). Spending an inordinate amount of time in a game can also have a damaging impact on the non-game environment, such as by resulting in conflicts within the family (34). In any event, it is clear that with the emergence of games with an infinite game duration, the problems of children and young people in their daily functioning, even though percentages are small, have increased (35, 36). Moreover, specific game genres seem more prone to problematic game behavior than others and this could be related to specific design choices, including reward mechanisms (37). This could potentially also mean that some game genres are less age appropriate for children when they are not contributing to their well-being, or that some game genres are inherently harder to make age appropriate because of design features that are an inseparable part of it. The World Health Organization added gaming disorder to its disease classification models in 2018 although this only applies to a small percentage of gamers (38). Moreover, with respect to some of the issues with gaming, such as excessive use, there are often other underlying social or emotional problems as well (25).

The incorporation of gambling elements into the design of games can also pose health effects. Children and young people are particularly susceptible to gambling because of their still evolving capacities and online gambling may therefore not be offered and advertised to children in some countries (39, 40). However, there is a diversity of ways in which loot boxes are currently regulated worldwide (41). Underlying this is the question of whether loot boxes lead to problem behavior similar to that seen in gambling, and are a reason to strictly regulate gambling and prohibit it for children. There are indeed indications that loot boxes cause problem gambling in adolescents and game companies can actually profit from adolescents with gambling problems (42, 43) and even if conclusive evidence is missing the precautionary principle may still demand regulatory intervention to at least protect children when there is potential harm (41, 44, 45).

Although we have not focused on marketing as a persuasive strategy to steer behavior through design specifically, marketing in games can have an impact on children's development and health. This is particularly the case in so-called advergames (games in which the commercial message is completely interwoven with game play and is often no longer recognizable to the gamer) (21, 46). Advergames are particularly notorious for promoting unhealthy food brands (46). It is generally accepted that advertising can have negative side effects on children in terms of encouraging materialistic values, unhealthy lifestyles and parent-child conflicts (47).

### Avoid Exploitative Game Design

We have shown that evolving revenue models of the game industry have had a substantial impact on game design and, in particular, have led to design choices that influence the behavior of gamers in such a way as to generate more profit. Although it should of course be possible for game companies to make money with their games, there are certain design choices that can be qualified as forms of exploitative design that in relation to children should not be used. This follows from their right to protection from economic exploitation as laid down in Article 32 CRC. This right aims to, among others, protect children from being abused by unfair methods of gaining commercial advantage (48). Such unfair methods may include deception and manipulation of children in ways that they are not aware of (49), as well as other forms of potentially exploitative design, i.e., design primarily or exclusively for an economic purpose. Such methods are considered to violate the “human dignity of the child or the harmonious development of the child's personality” (50). The Committee on the Rights of the Child acknowledges that particularly “Reaching adolescence can mean exposure to a range of risks reinforced or exacerbated by the digital environment, including [...] economic exploitation” (51). In essence the right entails that while companies can pursue economic goals with their games, children's vulnerabilities should not be exploited for profit. In our view, the impact of exploitative design is however broader than just for adolescents, but certainly also affects younger gamers.

Protection from economic exploitation of children is considered important because it does not contribute to the child's well-being and healthy development and can even be harmful, economically, socially and emotionally (48). Given the shift toward in-game monetization models and the commercial interests that go with it, online economic exploitation of children is increasingly becoming a focus of attention (48, 52). Exploitation of children can take three, intertwined forms: (1) economic exploitation of children's personal data (see also next section), (2) economic exploitation of children's cognitive development (e.g., manipulating economic choices through marketing with respect to in-app purchases and in advergames that may increase the likelihood of unhealthy choices, such as buying candy offline), and (3) economic exploitation by having children engage in economic activities (think eSports and child influencers) if they are harmful to them (48, 53). All these forms of economic exploitation are relevant in relation to game design.

Economic exploitation in gaming more specifically includes ‘dark patterns' which are intentionally misleading interfaces that unwittingly trick users into, for example, spending money or sharing more personal data than they would have done if it had been a conscious choice. Specific examples are hiding the actual economic value in (constantly changing) in-game currencies, automated profiling of users for optimizing profitable in-game behavior, and forcing users into in-app purchases to boost their gaming performance. Esports, as a new form of work which also allows children to earn money, is another development that is starting to receive more notice (53) and may be considered a new form of child work. Another way for children to make money are Twitch streamer where viewers can make donations while the gamer is playing. Children may also be faced with special requests, for example, to display tempting behavior (54, 55). In the latter case, economic exploitation may coincide with sexual exploitation of the child. Making money by children is not necessarily economic exploitation but work by children is subject to legal restrictions in many countries.

To a certain extent, the protection against economic exploitation of children is regulated in consumer law and data protection law. Some forms of manipulation are considered unfair commercial practices, when they push consumers, i.e., gamers in our case, to make decisions they would not have taken otherwise. Such practices include business activities that violate the requirements of professional diligence and materially distort the ability of the average consumer to make an informed decision (Article 5 Unfair Commercial Practices Directive). A distinction is made between misleading commercial practices and aggressive commercial practices. Gameplay and commercial messages that are intertwined and indistinguishable from each other and intended to encourage gamers to pay for access to premium content or features are, e.g., regarded as misleading commercial practices by the UK Office for Fair Trading (23). Games that suggest that a particular feature is scarcer than it actually is, or that suggest that gamers are somehow inferior if they don't do something that requires a purchase, may be examples of aggressive business practices (23).

While some commercial practices are easier to recognize from the outside, design practices to enhance monetization that are “inside” or coded in the game are more complicated to address. Activision's patent, filed in 2015, on a type of monetised matchmaking is a good example (14). It describes a system that “may match a more expert/marquee player with a junior player to encourage the junior player to make game-related purchases of items possessed/used by the marquee player.” This is not a matchmaking system designed to make a game more fun, instead its purpose is solely to increase monetization. A further development of this type of monetised matchmaking is Activision's more recently filed patent. In 2019, a patent was filed for what Activision calls “skill-based matchmaking” or SBMM (56). A gamer's personal data, such as their skill level, items used regularly, their frequent locations in game, and their previous in-game purchases are all used to match players in such a way that they will more easily purchase in-game items. Such a type of matchmaking, designed to encourage microtransactions, might not necessarily be unfair for adult players with disposable income if the company is transparent about it. However, for children that are both more easily influenced by these tactics and have less disposable income, such a type of system might fundamentally alter their gameplay experience and be unfair. It is therefore necessary to take all parties involved into account when determining whether or not a certain practice is fair, as there is no universal standard.

Different business models raise particular challenges in relation to economic exploitation and the (un)fairness of commercial practices more specifically. Although the application is based on national implementations of the Unfair Commercial Practices Directive in the European Union, we will give some considerations with respect to the business models that exist in games that may be relevant in determining whether practices are (un)fair, analyzing respectively subscription models/games as service, free-to-play games, microtransactions, in-game currencies, loot boxes, in-game advertising and out-of-game revenue models.

#### Subscription Models, Games as a Service

The objective of a subscription based business model is to retain customers in order to secure a recurring revenue. Since customer relationships are important to the success of a subscription-based business model, game developers are encouraged to ensure that the game remains attractive for the gamer to continue subscribing. However, the game design should not make it difficult for gamers to end the subscription to the game. Any design that makes it noticeably more difficult for gamers to withdraw from the game may amount to an aggressive business practice (57). This could be the case when characters or equipment are lost upon termination of the subscription. Also, the loss of access to friend groups and the online social life in the game environment could have a huge impact on gamers, especially teenagers, and deter them from canceling a subscription.

#### Free-to-Play Games

Offering an app or game for free while incurring additional costs (e.g., in-app purchases not clearly identified) is considered a misleading commercial practice. Essential information for a consumer's decision to play, download or subscribe to a game (such as the cost) must be provided clearly, transparently and accurately (23). Before consumers buy or download a game, a provider must inform them about in-game purchases and must clearly indicate which parts of the game are free or not (58). A general disclosure of the presence of in-app purchases in games might not be sufficient for the consumer to make an informed decision. In Super Mario Run, for example, the consumer is informed of the presence of in-app purchases. But it is not clearly specified that when you are prompted to pay € 10,99 to unlock the other worlds that this only applies to the “World Tour Mode” (and not to the “Rally Mode”). The vanity content is still up for purchase and can only be accessed through “? bonus blocks,” buying or gaining coins and through rally tickets (which one must purchase). In addition, while the “number of playable characters” will increase, not all characters are unlocked.

Moreover, “free-to-play” games, in which no monetary payment is required, may be a misleading commercial practice when there is no transparency regarding the actual “cost” of accessing the game. The ban on calling something “free” when it is not, is based on the idea that consumers expect a “free” claim to be just that, i.e., they get something without giving money in exchange (22). However, there is a growing awareness that personal data has economic value and is the price of entry for digital content and indeed personal data, including consumer preference, are being sold to third parties (22, 57). When the collection and use of the gamer's data are part of the main monetization strategy of the game, the insufficient provision of information regarding this practice (and basically hiding the commercial intent) is problematic as it does not allow the gamer to take an informed decision on whether to play the game or not. In addition, there is the question of whether the processing of personal data is done lawfully at all. If the provision of a service, i.e., playing a game, is conditional upon consent for the processing of personal data that is not necessary for said provision then consent is not considered to be freely, and therefore lawfully, given (Article 7(3) GDPR). A violation of EU data protection law must be considered when assessing the overall (un)fairness of commercial practices (22). In the case of children in particular, the conditions are stricter under both consumer and data protection law.

#### Microtransactions

Freemium business models have strong incentives to design a game in a manner which maximizes microtransactions. Various techniques are used to increase gamers' engagement and encourage the gamer to spend money on the game. Some examples of techniques used to stimulate purchases and trigger impulse purchases include the use of offers that are valid for a limited time, price personalization, and algorithms that determine the best sales strategy (58). Techniques used to encourage microtransactions sales that pressure gamers to the point where they have a hard time making a well-considered decision are considered to be the exercise of unacceptable pressure (and therefore an aggressive commercial practice) (58). Consider, as an example, the conversion between real money value and various token structures with complex exchange rates, which often complicate the assignment of value to in-game currency and create cognitive load. The use of algorithms to exploit psychological vulnerabilities in groups of players, such as children, to determine whether and when an offer can be made is considered to be an aggressive commercial practice (58). In addition, it seems problematic to use nudge techniques to exploit subconscious processes, such as cognitive biases (e.g., loss aversion) or associations between certain colors of imagery (“trigger our preference for shiny buttons over gray ones”). Moreover, games that target or appeal to children should not directly encourage children to purchase items in a game. This includes pressuring a child to buy the game directly or asking them to persuade an adult to buy items for them. Examples include “buy now” or “upgrade now.” When assessing marketing directed at children, due consideration should be given to the way messages are presented and of the context of those messages (41).

Game design patterns in which gamers are tricked into spending more money than they expected or anticipated occur in various forms (6). For example, gamers are deliberately and continuously confronted with frustrations and frictions (e.g., extremely long waits) that can be eliminated by small transactions. “Pay to skip” is a pattern where you can progress in a game or take a shortcut in exchange for a payment. A particularly aggressive version of the “pay to skip” pattern occurs when the gamer's ability to play effectively steadily declines until payment is required to progress in a meaningful way (6). An Android game, Replica Island, tracked players' frustration levels. This tracking can be used to make the game more enjoyable, but it can also be used to balance frustration such that the player is more inclined to make a purchase (59).

Monetised rivalries or “pay to win” patterns take advantage of the gamers' competitiveness, encouraging them—*via* behavioral game design that has nothing to do with fun and creating a intrinsically meaningful game-to spend money they would not otherwise have spent in order to achieve in-game status such as a high place on the leaderboard (6). The problem is not that companies engage in marketing or enable in-app purchases in games, but that the strategies they use are not always transparent and may manipulate behavior in ways that go beyond mere encouragement.

#### In-game Currencies

Many games use their own virtual currency. This currency can be earned in some games (usually at a slow pace, by grinding) or purchased with real money. Examples of these currencies include Vbucks in Fortnite, Robux in Roblox and FIFA (FUT) coins in FIFA. Gamers tend to spend virtual money more easily, as the association with real money disappears and players become unaware of the true cost of certain in-game items. For example, in Fortnite, premium currency can be purchased with real money, but the exchange rate must be calculated manually and can only be calculated with the information on the screen where one buys it. This kind of designed ambiguity intended to make it easier for consumers to spend their premium currency, as they might not become aware of its true value. Therefore, since the price of the product is one of the most important features of a product, it should be included in any invitation to purchase. It is not enough to state the price in the currency of the game. Games must also state the cost of the product in euros with each offer (58). Deliberately hiding the actual price of currency, either by not stating it, or by a design pattern that causes gamers to lose track of monetary value and thus that they are actually using real money, is an omission of essential pre-contractual (price) information and is considered a misleading commercial practice. In-game currencies are also reported by players as a problematic design feature (60).

#### Loot Boxes

Loot boxes contain one or more virtual items that vary in value or rarity and that gamers can buy or win. Players do not know what is in the loot box until they open it, and usually the rewards are awarded randomly. A player can sometimes unlock loot boxes without additional payment by, for example, completing certain in-game tasks. Alternatively, players can purchase loot boxes with real money, or in-game currency. The invitation to purchase a loot box must include not only the cost of the loot box in euros, but also the chance of obtaining a rare item. If players can sell the contents of the loot boxes, gambling law applies. But because the gambling element of loot boxes plays into the player's vulnerability, games may violate unfair commercial practices law even if the content cannot be traded (58). The gambling authority in the Netherlands has indicated that the loot boxes mechanisms employed in several popular games (including: FIFA) are likely legally considered as gambling and therefore in violation of Dutch law (as offering online games of chance / gambling without a license is prohibited) (61).

#### In-game Advertisements and Product Placement

Gameplay and commercial messages that are intertwined and indistinguishable from each other, encouraging gamers to make transactions, can be misleading and result in unfair commercial practices. This could be the case when a game uses similar language to describe the exchange of in-game currency for game features and the purchase of in-game currency for real money. Another example is when there is an indistinguishable transition between gameplay and the store and the purchase process is initiated without making it clear that an actual purchase must be made in order to continue with the game (23).

Games in which the commercial message is immersed in the digital game content *via* brand or product placement, however, are more complex. Unlike traditional forms of advertising, new forms of marketing are becoming increasingly integrated into the game experience and more personalized (21). If an advergame is targeted at children without providing information about the commercial nature of the game, this could be considered an omission and thus an unfair commercial practice (21).

#### Out-of-Game Revenue Models

Economic exploitation of children can also occur both in-game and out-of-game by having children engage in economic activities such as streaming (child influencers) and eSports. However, there are currently no laws in the EU that protect children from this form of economic exploitation. The Unfair Commercial Practices Directive and the revised Audiovisual Media Services Directive contain several protections for child-viewers of the video streams or eSports tournaments, “usually focused on identifying the commercial nature of videos or prohibiting direct exhortations to children,” but do not consider the position of the child-influencer (53). Both influencers and eSports participants invest a lot of time to be successful and can be under a lot of pressure to perform. However, the protection of children from harmful or emotionally demanding work may be limited to employment relations, which is not the case in this new form of work. Despite the potential harmful effects of influencer work and other digital work (like eSports), this form of child work remains unregulated and leaving children largely unprotected but for their parents setting restrictions (53).

### Limits to Data-Driven Game Design

The move toward data-driven revenue models that track gamers' behavior and use it to identify profitably interesting gamers and for targeted marketing raises data protection issues. In the case of children, EU data protection law, i.e., the General Data Protection Regulation, provides an even higher level of protection which is certainly more difficult to comply with in the case of data-driven business models. As indicated in the previous section, the excessive use of personal data can be considered a form of economic exploitation of children (48).

#### Data Driven Gaming and AI

Data-driven services include, for example, games that operate with personalized ads, that is, ads that are targeted to gamers based on interests, characteristics, and sometimes vulnerabilities derived from their behavior and other personal data. The developments in the field of artificial intelligence that also have an impact on game design (e.g., because of the opportunities for monetisation) may further increase the data-driven nature of games and thus the potential to better exploit the vulnerabilities of gamers in monetisation schemes. An example cited earlier of monetised matchmaking (see Section Behavioral Design in Games and Revenue Models).

Here it is important to stress that not all use of AI in gaming is an example of a data-driven service. Computational algorithms influencing player experiences based on in-the-moment play data or long-term player data has a long-standing history in game play. Crash Bandicoot 2 (1997), for example, was one of the first games that featured Dynamic Difficulty Adjustment, where every time a player died, the game became a bit easier (62). Other games (e.g., Devil May Cry) adjust the playstyle of NPC's in consecutive levels on the basis of the player's playstyle, to ensure consistent difficulty (63). Algorithms are also used to support aiming in controller-based games to compensate for the lack of accuracy in controller input (64). An AI would be a certain algorithm that also has a self-learning factor, which makes the algorithm more efficient or better. These techniques are generally quite safe, given that they do not influence the player themselves, they only help the player slightly to make the game more fun.

AI becomes problematic when AI or computational algorithms are solely used for increasing profitability, not for increasing the actual quality or playability of the game. When the sole goal of the AI is profitability, the rights to protection against economic exploitation and health, especially for children, can easily be impacted. It should be noted that when the goal is mostly increasing playability or making the game more fun can have the unintended consequence of affecting health too. However, in this case, it is more difficult to tell if this is attributable to the designers or not and safeguards can more easily be implemented.

AI can grow even more problematic when player data is being used (excessively), as this allows for more efficient manipulation. Using player data, such as player performance data is not new. Games such as Chess have been using player performance data to calculate ELO points, a type of Chess rating to indicate skill level, even before computers were involved (65). Newer techniques to calculate player performance, such as TrueSkill^TM^ (66), infer individual skills within a team to predict skill and improve matchmaking accuracy. Video games also adjust difficulty dynamically based on player performance, e.g., the number and frequency of zombie waves in Left 4 Dead. The rationale behind these types of algorithms may be simple, if the difficulty is always being adjusted—not too difficult, not too hard—players are more incentivised to continue playing. However, a continuously adjusted difficulty in pay-to-win games might mean that frustration-levels are also continuously at the optimal point to encourage in-game purchases. Again, the technology becomes problematic once it is solely used for a profitability objective. It should, however, be noted that even if the only goal is making a game more fun or enjoyable, as an unintended side effect, the game could still facilitate high engagement and extreme time spending.

Unsupervised algorithms are heavily dependent on the data they are trained on, which might result in training mistakes that exclude individuals, for example when training with able bodied people, but exclude disabled players. Bias in data or incomplete data, can lead to an incomplete or biased AI (67). Another risk is the unethical extraction of information from player behavior, e.g., stealth assessment of cognitive function or even identity theft.

#### High Level of Protection for Children

Whenever personal data is processed as part of game play, be it simple data to sign up for a game or to enable data driven strategies as part of behavioral design in games, data protection law requires a high level of protection for children's personal data because children are considered particularly vulnerable when it comes to the processing of their personal data (68). The special position of children is due to their decreased ability to assess the risks and consequences of data processing (Recital 38 GDPR). In 2021, the UN Children's Rights Committee recognized that the right to privacy of children (Article 16 CRC) also includes a right to data protection (49).

To consider children and the higher-level protection of their personal data, it is important that game providers know which gamers are children. Self-declaration (i.e., stating your age of birth date) is the most common method used in games to verify gamers' age, and it can be circumvented quite easily. For children under a certain age, there is an incentive to declare a different, higher age, if otherwise they will be excluded from the game (69). The level of assurance of age verification is not only relevant from a data protection point of view, but also because in the case of children, stricter rules may exist from other points of view (welfare, health, harmful content and exploitative design etc). Moreover, high risk data processing, e.g., the processing of personal data for the purpose of profiling and the processing of children's personal data, requires high assurance age verification which means that more is required than self-declaration of age (69). Games that use data driven strategies as part of their game design must adhere to a higher level of protection for children. Under the GDPR, it is assumed that if a game is not restricted to 18+ and there is no evidence to the contrary that children play the game, the high level of protection awarded to children must be taken into account (68).

A number of initiatives have been developed to support technology designers and developers in implementing data protection law in an age appropriate way: i.e., the age appropriate design code (or Children's Code) in the UK (70), Fundamentals for child-oriented approach to data processing (draft) in Ireland (71), and the Code voor kinderrechten (Children's Rights Code) in the Netherlands (72). Moreover, the emergence of this type of initiative shows that the topic of taking into account children and the high level of protection that their personal data must enjoy is receiving increasing attention.

The high level of protection under EU data protection law limits the opportunities for data-driven game design in games used by children for several reasons. For instance, their data-driven nature raises specific issues in relation to children concerning the fairness and transparency of data processing, as well as the lawfulness of data processing in relation to consent and profiling of children.

#### Fairness

Fairness is an important principle in EU data protection law (Article 5 GDPR). The principle means that any power relationship that shows an imbalance-in this case, the relationship between game company and gamer-must be rebalanced. A good example of an asymmetric power relationship is the interpretative capabilities of a child and a privacy policy written by a team of lawyers. It can quite easily be seen how it is not fair to expect from a child to understand a complicated privacy policy that must explain complex data driven practices. This need for rebalancing is effect-based; of less relevance are the formal procedures of e.g., transparency of data processing; only the substantial mitigation of unfair imbalances can be called “fair” (73). In cases where the power imbalance or knowledge gap is greater, due to for example the monopolistic position of a company or the vulnerability of the gamer, the fairness principle could be said to apply more strictly as there is more to rebalance. With companies such as Electronic Arts, Activision Blizzard, and Ubisoft, the bargaining position of an individual is almost negligible. An individual would not be able to renegotiate the terms of service and privacy policy of Ubisoft.

The fairness principle is intended to ensure that the position of an individual and a company is such that there is no need to try and renegotiate, as the situation is already fair. Here, for example, there is a clear connection to the principle that only data that is necessary for a company's operation of services, in this case games, is processed (principle of data minimization; Article 5 GDPR) and such processing must also comply with the privacy by design principle. Both principles are at odds with data-driven design and, from a fairness point of view, this is even more problematic when processing personal data of children. Given the focus on power imbalances, it can therefore make a difference who the gamer is: an adult or a child; what is still fair with respect to adult gamers may not be fair with respect to children, or others that are vulnerable for that matter. For example, adults can consent to data processing for personalized marketing, whereas this is increasingly seen as undesirable in relation to children. This principle of fairness is also context-dependent; what is fair in effect differs in every situation and must be individually assessed. There is no universal standard, some data processing practices can be fair in certain situations and unfair in others. Moreover, what is considered unfair under data protection law can also be unfair under unfair commercial practices law, as we have seen in the previous section.

#### Transparency

The principle of fairness is closely linked to the principle of transparency because one way to make a power relationship more balanced is for an organization to be transparent about what they do with personal data. The principle of transparency therefore intends to ensure that full transparency of data practices and rights is ensured to gamers. In the case of children, this means that information relating to data processing must be comprehensible, recognizable, and accessible to them (Article 12 GDPR) (74). The principle goes beyond providing said information in a formal way and dictates that game companies must not take unjust advantage of their position by essentially keeping gamers in the dark about data practices with incomprehensible legal jargon or complex game design. This inextricably links transparency to the principle of fairness. The more complex data practices and the corresponding game design are, the harder it will be to explain them properly to gamers, especially if gamers are children. However, transparency is not a silver bullet to establish a balanced relationship-data practices can still be unfair even if a company is transparent about them in an accessible and understandable way.

Questions of transparency (and fairness) for gamers are raised by AI in game design because of the black box problem. The black box problem occurs when an AI is sufficiently complex that even the designers and computer scientists cannot fully trace every step in its decision-making (75). Additionally, it is unclear to the gamers themselves that AI manipulations and adjustments are behind their gaming experience. In Activision's skill-based matchmaking, we might know that the AI uses previous in-game purchases and frequently visited locations on the map to matchmake, but how these two data points correlate and affect the AI's decision is a mystery. This can have a detrimental impact on the transparency that is required by data protection law in case of automated decision-making and profiling with a significant impact on gamers' legal or economic situation, or personal health and well-being.

The use of AI can also become even more problematic when its sole purpose becomes monetization rather than game optimization. It is increasingly difficult to put into words how your data will be used, even complex privacy policies or privacy settings might not be adequate for this purpose in the future, let alone child-friendly ones. Simply put, the more complex AI's grow, the more reason there could be for a prohibition based on the inability to provide adequate transparency and information of data processing by the system.

All this touches on the legitimacy of data processing as well. Data driven practices require consent which must be informed.

#### Lawfulness: Consent

To process personal data lawfully, the game company must have a legal basis. The obvious legal bases for processing personal data in games all have specific requirements in the case of children (68). However, in the context of this contribution, it is particularly relevant that for data-driven digital services, including games, only consent provides a legitimate basis [Article 6 (1) (a) GDPR]. Consent can only be given by a person that has reached the age of digital consent and depending on their age, this is not the case for most children (Article 8 GDPR) (68). Children can consent to the processing of personal data from a certain age (16 in the Netherlands, but other EU member states may apply different ages). If the age of digital consent has not yet been reached, consent must be obtained from one of the parents or guardians. Therefore, data processing is potentially unlawful if consent is given by children who are too young to do so by law, which may happen if they can easily circumvent age verification mechanisms (69). Self-declaration is mostly used for age verification, which therefore does not provide sufficient assurance in this case because it can easily be circumvented. In this case, it does not matter if children give wrong information because age verification is a responsibility for companies that they must have organized at a demonstrably sufficient level of assurance. There are then two options: either the game company implements a demonstrably high level of age assurance or does not process personal data based on consent. Consent is the lawful ground only used if none of the other lawful grounds in the GDPR apply and is therefore only necessary for a small part of the data processing. However, this includes processing that is used for data-driven practices which can have the function of behavioral design for profit.

Moreover, consent is subject to strict conditions that apply regardless of the age of gamers (Article 7 GDPR). For instance, consent must be informed, which means that gamers must know what data processing they are consenting to. Designed obscurity immediately makes the choice less informed, i.e., the more complex the data processing, including the design of games that enable such data processing, the harder it is to explain to the gamer, especially when a child, and the less likely it is that consent is informed and therefore lawful. Besides the sheer complexity of data processing practices, individuals are often unable to make an informed decision due to information overload (76). A recent study shows this ineffectiveness of privacy policies and terms of services even further. In an experiment, 74% of participants did not even read or skim privacy policies and 98% of participants were unaware that the data of their first-born child was being monetised (77). In the case of children, again the GDPR explicitly requires that it must be understandable to them (Article 12 GDPR). Also, the consent process itself should not be designed as a dark pattern where the gamer is tricked into giving consent for invasive data processing when creating a game account while the privacy-friendly options are hidden in the settings of the account. All in all, consent is not a lawful basis that can easily apply to data-driven activities of game companies, certainly not with respect to children (69).

Box 1 **Example: Clash of Clans**

Clash of Clans, a game by Supercell, is an example in which the way consent is obtained is problematic and the valid use of other legal grounds for the processing of personal data are questionable.

Upon first installing the app, there is only one option to start to play the game: accept the terms of service and the privacy policy or don't play the game. Although mentioned, both documents are not explicitly presented to the user. The terms of service are available to consult by clicking on the button “terms”, but no separate link or button is provided to consult the privacy policy. Even though Supercell's privacy policy is also incorporated in their terms of service, there is no way to check quickly and simply what they use a person's data for, how it is used, or why they use it.

According to Supercell's privacy policy (Effective date: November 15th, 2021) data may be processed for “additional purposes” with the player's consent. What these additional purposes are, is not specified. In any case the acceptance of the privacy policy by clicking “ok” is not consent under GDPR and one of the reasons is that it is not informed (the policy is not presented before clicking “ok”).

#### Lawfulness: Profiling

The underlying process of data-driven revenue models is the automated profiling of, in our case, gamers to find out their interests, characteristics and potentially also vulnerabilities. Based on behavioral and metadata, algorithms create so-called inferred data, which can potentially contain very sensitive information, such as medical conditions, sexual preferences or political affiliations (15), and be used for targeting groups of individuals or even individuals. The goal is to develop models of customer groups that drive revenue and make decisions around those groups. Moreover, targeted ads are used in games financed through ads. Zynga (Farmville), for example, has their own ad-network where they also advertise to their own customers (78, 79), e.g., by identifying which game types they like so that they can recommend others. Essentially, it is our impression that the majority of game companies profile, even the smallest studios, because they want to know who buys their products. Profiling of children by capitalizing on their personal data, and particularly on their inferred personality traits, preferences, gaming behavior or vulnerabilities, can amount to a form of exploitative design (48).

The GDPR prohibits automated profiling that has a legal effect or similarly significant effect on gamers (Article 22 GDPR). A legal effect can be present when automated profiling pushes children toward in-game purchases they would not have otherwise made. Similarly significant effects may include detrimental consequences for a gamer's physical or mental health or well-being, e.g., when automated profiling discloses a person's vulnerabilities in terms of impulsive gaming or in-game spending behavior. Being economically exploited through AI optimisation could also be considered another significant effect and children can be more vulnerable in this respect. The same applies to marketing aimed at children, as it is known that it can have negative effects on them (21, 47). Effects that can possibly be strengthened by forms of marketing directed at children's vulnerabilities or marketing that is no longer recognizable as such (21, 47).

Although the GDPR has exceptions to the prohibition of automated profiling, it is generally assumed that these exceptions generally do not apply to children. Game companies must therefore avoid profiling children unless it is in their best interest (48, 68). This means that AI based automated profiling must be avoided in the case of children unless it is in their best interest. Profiling could be in their best interest if it makes the gaming experience more enjoyable or challenging without compromising their well-being. Even then, however, it remains an activity for which it is likely that there must be consent from the gamer or their parents to be lawful: as we showed earlier, that is a difficult task for gaming companies.

In this respect it is interesting that the draft EU AI Regulation proposes to prohibit certain AI practices including those that “deploys subliminal techniques beyond a person's consciousness in order to materially distort a person's behavior” and “exploits any of the vulnerabilities of a specific group of persons due to their age, physical or mental disability, in order to materially distort the behavior of a person related to that group” [draft Article 5 (1) (a) and (b)], the latter of which clearly also focuses on children. However, in both cases an important limitation would be the requirement of physical or psychological harm (80). Although harm to the well-being of children can certainly occur, as we saw earlier, it can be difficult to prove. Moreover, in the case of children, especially younger ones, it is assumed that a precautionary approach (also called the better safe than sorry approach) must be adopted with respect to children if there are indications of harm but no definite proof of harm (21, 49, 81). Moreover, these provisions ignore the question of whether economic exploitation in the case of children is not inherently unfair, whether there is visible or provable harm or not. The UN Committee on the Rights of the Child is concerned about the commercialization of children's lives, online and offline, and indicates that some practices, including profiling for commercial purposes, immersive advertising and advertising in virtual and augmented reality environments, should be prohibited (2, 49).

## Conclusions

Attention to children's rights in relation to the design and use of digital technologies is growing. This includes specific issues related to *behavioral design* in games: design practices that steer gamers and their behavior in a variety of ways, both positively and negatively. Recently with the emergence of new gaming business models, we are seeing behavioral design take on forms that are particularly worrying for children and interfere with their rights. Although the UN Children's Rights Committee does not specifically focus on behavioral design in gaming in their recent general comment on children's rights and digital technologies, it pays attention to design strategies also applied in games:

“*The digital environment includes businesses that rely financially on processing personal data to target revenue-generating or paid-for content, and such processes intentionally and unintentionally affect the digital experiences of children. Many of those processes involve multiple commercial partners, creating a supply chain of commercial activity and the processing of personal data that may result in violations or abuses of children's rights, including through advertising design features that anticipate and guide a child's actions toward more extreme content, automated notifications that can interrupt sleep or the use of a child's personal information or location to target potentially harmful commercially driven content”* (49).

Some of these practices, such as profiling of children for commercial purposes, neuromarketing, emotional analytics, immersive advertising and advertising in virtual and augmented reality environments, must be prohibited, according to the Committee (49), but in any case the best interest of the child must be a primary consideration when designing digital technologies that are used by children.

It is important to consider the developments and rights discussed here holistically. Firstly, for unraveling fully how gaming and game design in their multifaceted nature can have an impact on children's rights and therefore require attention. But also, because children's rights are interwoven, and it is only when they are considered together that the impact can be properly assessed. It is important to keep in mind that children have different kinds of rights, such as protection and participation rights, that need to be balanced when addressing the negative impact of behavioral design in games. While it is important to ensure the protection of children from economic exploitation through strict enforcement of existing laws, this should not be at the expense of their right to play and relax, to socialize with others in games and to take advantage of the huge potential of gaming for creativity, education, and a sense of achievement.

The UN Children's Rights Committee sees a responsibility for the industry to implement children's rights and calls for the development of industry codes and design standards in line with children's rights (49). Such codes and standards can make an important contribution to the lawful, ethical, and responsible game design, if they are legally enforceable, and enforced, or become fully implemented and part of common business practice in other ways. In this contribution, we have shown how game design can contribute to a joyful, sociable, inclusive, and healthy game environment, qualities of play that can be translated into design practices for age-appropriate games. Another recommendation put forward by the Committee is to make the performance of child rights impact assessments by company's compulsory and disclose them to the public. Such impact assessments should express all children's rights in a coherent manner and identify mitigating measures for possible negative impacts of game design on children of different ages. Sharing them can stimulate the learning capacity of the gaming industry in this area.

These recommendations will only become more important in a future where AI and immersive technologies such as augmented reality and virtual reality seem to play an increasingly important role. The increasingly immersive and realistic nature of games is thus greatly enhanced, which can both contribute to the enjoyment of gaming and also raise new questions regarding the well-being of gamers and the ethical and legal nature of game design. It is important to, besides assessing the potential impact of these technological developments, involve gamers in time and to hear from them-from young to old-what they want and expect from these new game environments and experiences, and what they are concerned about.

## Data Availability Statement

The original contributions presented in the study are included in the article/supplementary material, further inquiries can be directed to the corresponding author.

## Author Contributions

SHo: involved in all parts of the article, writing of the first draft of the full article, and involved in final edit. SHi: contributing author data protection. SO: contributing author unfair commercial practices. MB: contributing expertise on revenue models and game design and involved in final edit. AR: contributing expertise on revenue models and impact of game design and involved in final edit. All authors contributed to the article and approved the submitted version.

## Funding

This research was carried out as part of a project on behavioral design in video games that was funded by the Ministry of the Interior and Kingdom Relations, the Netherlands and took place in the first half of 2021. This article is an adaptation of that research.

## Conflict of Interest

The authors declare that the research was conducted in the absence of any commercial or financial relationships that could be construed as a potential conflict of interest.

## Publisher's Note

All claims expressed in this article are solely those of the authors and do not necessarily represent those of their affiliated organizations, or those of the publisher, the editors and the reviewers. Any product that may be evaluated in this article, or claim that may be made by its manufacturer, is not guaranteed or endorsed by the publisher.

## Footnotes

^1^In this article “gaming” specifically refers to video gaming and digital gaming; in a similar vein games means video games or digital games.

^2^Loot boxes contain one or more virtual items that vary in value or rarity and that gamers can buy or win. Players do not know what is in the loot box until they open it, and usually the rewards are awarded randomly. A player can unlock loot boxes without additional payment by, for example, completing certain in-game tasks. Alternatively, players can purchase loot boxes with real money, or in-game currency.

^3^A series of educational games about pioneer life in the 19th century, the first of which was released in 1971. Currently it is published by Gameloft in the Apple Arcade; see https://www.gameloft.com/game/the-oregon-trail.

^4^A game which takes place at the time of the French Revolution in which the player is a judge; see http://we-the-revolution.com/.

^5^A grand strategy game set in Europe from the late Middle Ages to the early modern period; see https://www.paradoxinteractive.com/games/europa-universalis-iv.

^6^See http://www.positech.co.uk/democracy4/.

^7^A puzzle game in which the player, as an electronics engineer, must create products by building circuits and writing code to make them work; see https://www.zachtronics.com/shenzhen-io/.

^8^A sandbox game for programmers; see https://screeps.com/.

^9^A very popular sandbox game that shows a blocky 3D world with opportunities for e.g. building structures and experiencing adventures; see https://www.minecraft.net.

^10^An adventure sandbox game; see https://terraria.org.
